# A systematic review and meta-analysis on the association between PM_2.5_ exposure and increased influenza risk

**DOI:** 10.3389/fepid.2025.1475141

**Published:** 2025-04-11

**Authors:** Ava Orr, Rebekah L. Kendall, Zeina Jaffar, Jon Graham, Christopher T. Migliaccio, Jonathon Knudson, Curtis Noonan, Erin L. Landguth

**Affiliations:** ^1^ Center for Environmental Health Sciences, Biomedical & Pharmaceutical Sciences, University of Montana, Missoula, MT, United States; ^2^ Center for Population Health Research, School of Public and Community Health Sciences, University of Montana, Missoula, MT, United States; ^3^ Mathematical Sciences, University of Montana, Missoula, MT, United States

**Keywords:** fine particulate matter, lag effects, respiratory viral, infections, influenza

## Abstract

**Introduction:**

This systematic review and meta-analysis investigate the relationship between PM_2.5_ exposure and increased influenza risk (e.g., increased hospital admissions, confirmed influenza cases), synthesizing previous findings related to pollutant effects and exposure durations.

**Methods:**

We searched PubMed, Web of Science, and Scopus for relevant studies up to 1 January 2010, following Preferred Reporting Items for Systematic reviews and Meta-Analysis (PRISMA) guidelines for selection and analysis.

**Results:**

Our review included 16 studies and found that a 10 μg/m^3^ increase in daily PM_2.5_ levels was associated with an increase of 1.5% rise in influenza risk (95% CI: 0.08%, 2.2%), with significant variations across different temperatures and lag times post-exposure. The analysis revealed heightened risks, with the most significant increases observed under extreme temperature conditions. Specifically, colder conditions were associated with a 14.2% increase in risk (RR = 14.2%, 95% CI: 3.5%, 24.9%), while warmer conditions showed the highest increase, with a 29.4% rise in risk (RR = 29.4%, 95% CI: 7.8%, 50.9%). Additionally, adults aged 18–64 were notably affected (RR = 4%, 95% CI: 2.9%, 5.1%).

**Discussion:**

These results highlight PM_2.5_'s potential to impair immune responses, increasing flu susceptibility. Despite clear evidence of PM_2.5_'s impact on flu risk, gaps remain concerning exposure timing and climate effects. Future research should broaden to diverse regions and populations to deepen understanding and inform public health strategies.

## Introduction

Influenza, an acute viral respiratory infection, has long posed a substantial global health challenge with approximately 1 billion individuals falling victim to the disease and an annual death toll ranging from 290,000 to 650,000 worldwide (1). The persistent threat of influenza is not only confined to its annual toll but also extends to the potential of a catastrophic pandemic. Drawing parallels to the 1918 Spanish flu, which resulted in a devastating death toll of between 20 and 50 million individuals, the WHO has warned that the emergence of a similar pandemic is not a question of “if” but “when” (2). Historical and contemporary studies indicate that the ever-evolving nature of influenza viruses, coupled with increased interactions between humans and animals, significantly raise the risk of a new pandemic strain emerging. These historical along with recent pandemics serve as stark reminders of the potential impacts and that advanced surveillance and preparation strategies are essential for mitigation (3).

Seasonal flu affects all individuals in every county, but specific populations are at higher risk, including children, the elderly, pregnant women, and individuals with chronic medical conditions, as well as groups with greater risk factors for health inequities (4). Recent research has expanded the understanding of external factors influencing influenza dynamics. Climate change and environmental variables, such as colder temperatures, drier climates, and air pollutants, particularly PM_2.5_, have been identified as critical determinants in influenza transmission (5–8).

Accumulating epidemiological evidence indicates an association between elevated PM_2.5_ levels and increased respiratory health risks, including heightened susceptibility to influenza infection. Previous studies have consistently reported a positive relationship bewtween PM_2.5_ exposure and influenza incidence, although results vary bygeographical region, population demographics, and exposure timing (9, 10). Despite these individual findings, a notable gap remains, as no systematic review or meta-analysis has yet comprehensively summarized existing evidence to clarify the strength, timing, and consistency of the PM_2.5_ influenza association across different settings and populations. Addressing this gap is essential to provide robust evidence for public health strategies aimed at mitigating influenza risks associated with air pollution.

This study offered novelty by systematically synthesizing existing epidemiological evidence on the relationship between PM_2.5_ exposure and influenza, particularly within the context of environmental changes driven by climate change and rising air pollution levels. By conducting a systematic review and meta-analysis, this research identified critical patterns, highlighted differences across populations, and clarified the timing of PM_2.5_ exposure impacts. These insights are essential for public health interventions, guiding policy decisions, and supporting proactive measures against the combined threats posed by influenza, air pollution, and climate change. Future research must adopt a balanced approach, integrating associative studies—examining epidemiological relationships—and mechanistic studies that explore underlying biological pathways. Clarifying both how and why PM_2.5_ affects influenza susceptibility will enable public health strategies to be tailored specifically to diverse global contexts, accounting for regional variations in air pollution sources, population vulnerabilities, and environmental changes.

## Methods

### Search strategy

The systematic review and meta-analysis were performed in accordance with the Preferred Reporting Items for Systematic Reviews and Meta-Analyses (PRISMA) guidelines and checklist in the Supplementary File (11) and complies with the recommendations of Meta-analysis of Observational Studies in Epidemiology (12).

**Figure 1:**
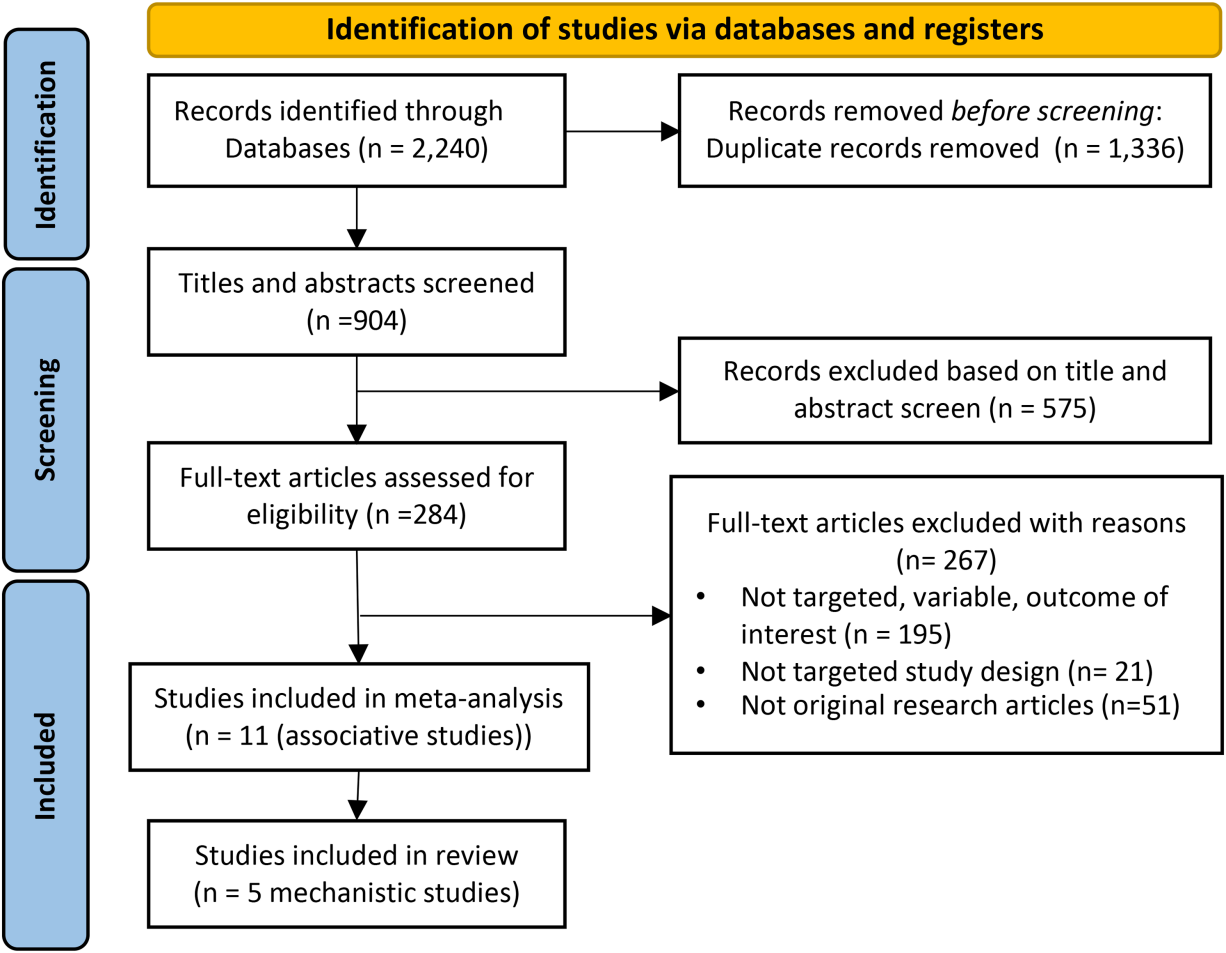
PRISMA flow diagram of study selection. Initially, 2,240 records were identified. Post-duplicate removal, 904 records were screened, leading to 575 being excluded after title and abstract review. Further assessment of 284 full-text articles resulted in 267 exclusions due to various reasons, leaving 16 studies (11 associative and 5 mechanistic) included in the final review.

This review focused specifically on PM_2.5_ due to its well-documented impact on respiratory health and its widespread monitoring, which ensured consistency across studies for a precise meta-analysis. Including other particulate sizes (e.g., PM10) would have added variability, as they differ in measurement and health impacts. We excluded influenza-like illness (ILI) studies to avoid conflating outcomes with non-influenza cases, focusing only on confirmed influenza results. Three electronic databases (PubMed, Scopus, and Web of Science) were systematically searched to find relevant studies in English between the time frame from January 1, 2010 to September 30, 2023, a time frame chosen to avoid overlap with previous reviews. Flow diagram for study selections are detailed in Figure 1. Briefly, a combination of keywords and search terms that included, but were not limited to, the following: “Influenza PM_2.5_”, “Influenza Air Pollution”, “Particulate Matter Influenza”, “Short Lag Influenza PM_2.5_”, and “Long Lag Influenza PM_2.5_.” Additionally, studies that provided mechanistic insights into the connection between PM_2.5_ and influenza were included. The results were then restricted to studies of humans. In addition, the reference lists of all included articles and pertinent reviews were also screened manually to identify more studies.

## Inclusion and exclusion criteria

The studies included had to meet the following inclusion criteria grouped according to the population-exposure-comparator-outcome (PECO) framework (13, 14). Potential sources of bias in this systematic review include publication bias (studies with positive findings are more likely to be published), heterogeneity in study design and exposure assessment methods, and variations in the measurement of influenza incidence across different studies.

• Population—population-based studies with no restriction on sex, age, or region
• Exposures—studies of short-term (0–7 days) or long-term (+7 days) exposures to any PM_2.5_
• Comparators—studies with clearly defined non-exposed or lower-exposure comparison groups
• Outcomes—studies assessing the incidence of influenza, measured by infection rates.
• Studies published in a peer-reviewed journal with full text available.

Studies with the following descriptions were excluded:

• Population—nonhuman studies (e.g., animal studies)
• Exposures—studies focused not on the health effects caused by PM_2.5_
• Outcomes—studies without an assessment of human health outcome

### Study selection

All studies included in the final review or synthesis were identified in several stages, beginning with automation tools, including a Python web scraping script using package “Biopython”, were employed initially to pull all relevant articles based on keywords, duplicates were then (15). Then, (A.O. and R.L.K.) independently reviewed the titles and abstracts of the remaining studies to remove obviously irrelevant articles. Finally, the remaining studies were screened for full texts for eligibility. Any uncertainty and disagreement were solved by discussion between the two reviewers.

### Data extraction

For each eligible study, the following information was extracted: first authors and publication year, study period, setting, design and population, sample size, exposure assessment method, exposure window, outcome, statistical method, and effect estimates (i.e., the effect estimates of influenza risk related to PM_2.5_ exposures). We collected data from sub studies within each eligible paper. Each study ran multiple models, often exploring different exposure windows, population subgroups, or analytical methods. Two authors (A.O. and R.L.K.) independently performed the procedure, and any conflict was discussed between the two reviewers.

### Study quality assessment

We assessed study quality using the Newcastle-Ottawa Scale (NOS) for non-randomized studies in meta-analyses (16). Discrepancies were resolved through discussions to ensure a rigorous evaluation. The NOS tool, applied manually, considered factors such as study group selection, comparability, and outcome assessment. This dual-reviewer approach aimed to provide a comprehensive and robust assessment of bias risk, contributing to the overall quality of the systematic review. No automation tools were used in the risk of bias assessment. The NOS uses the star system with a maximum nine stars and evaluates three domains with a total of eight items: the selection of the study groups, the comparability of the groups, and the ascertainment of the outcome of interest. The quality of observational studies was categorized based on the total stars received: seven to nine stars denoted high quality, five to six stars indicated moderate quality, and four or fewer stars were indicative of low quality (Supplementary Table S2).

### Statistical analysis

All studies included substudies, which allowed us to subgroup the results into different categories such as lags, age groups, and other relevant stratifications. Data for these subgroups were extracted from the results sections of each paper. The extraction process varied between studies, with necessary conversions performed as described below.

To uniformly measure the effects of PM_2.5_ exposures on influenza risk, effect estimates of relative risks (RR) (or similar measure) and their corresponding 95% confidence intervals (CI) were gathered from each study. It is noted that the availability of RRs across all studies was not uniform, as certain studies presented data in terms of excess rates or percent changes. To address this difference, percent change was converted to RR using the formula RR = 1 + Percent Change/100 and excess rate was converted by RR = Excess Rate + 1, ensuring a standardized and comparable metric for analysis.

Random- or fixed-effects meta-analysis was conducted on the extracted RR for each study to quantify the combined effects of PM_2.5_ exposure on influenza risk using R software (Version 4.3.2; R Development Core Team) “metafor” and “meta” packages (17, 18). To address the degree of heterogeneity within the included articles, we used the Q statistic-based *χ*^2^ test and *I*^2^ statistic by the Cochrane guideline (19). Low, moderate, and high hetergeneity were considered when *I*^2^ was less than 30%, between 30% to 75%, and exceeding 75%, respectively. A random-effects meta-analysis model was used. Publication bias was examined for the effect of PM_2.5_ exposure through a combination of funnel plots and the Egger's test.

To identify the source of heterogeneity, we also conducted a subgroup analysis based on sample size, patient ages, seasonality, and different lag exposure periods, allowing for the dissection of various sources within the collected data. Based on the included articles, we divided sample size into 6 groups: NA, <1 k, 1 k–10 k, 11 k–25 k, 30 k–90 k, 100 k; Age into 3 groups: <18, 18–64, >64, with a final group including “all” ages; Season (based on temperature) into 2 groups: Hot (24.2 °C–32.9 °C) and Cold (−4.5 °C–10.1 °C); Lagged exposures into 5 groups: “0–1”, “2–3”, “4–7”, “0–14”, “8–31”.

In the synthesis of mechanistic outcomes, quantitative analyses were not conducted due to variations in methods across the studies. Instead, a narrative overview was carried out, characterizing the mechanisms involved in immune responses triggered by PM_2.5_ leading to increased risk for infection of influenza.

## Results

The initial search yielded 2,240 records of which 1,336 were removed due to duplication. The remaining 859 were screened with 575 being further removed based on a review of the title and abstract. A total of 284 citations were fully screened. Consequently, a total of 16 studies were included in this systematic review and meta-analysis: 11 studies for the meta-analysis with another 5 that focused on the mechanistic underpinnings of the association between PM_2.5_ exposure and influenza (see Figure 1 and Supplementary Figure S2).

### Characteristics of the studies

The selected studies included different study designs and statistical methods: one case-crossover analysis (20), three studies employed distributed lag nonlinear models within their respective design (21–23), two studies utilized generalized additive models (24, 25), two applied random forest models (26, 27), two used regression models (28, 29), and one time series model (30). Each study had its own sub studies, often dividing the analysis by factors such as age, gender, temperature, and other demographic or environmental variables to assess subgroup-specific impacts. This allowed for detailed stratifications and comparisons, shedding light on how different population segments—children, the elderly, or those living in higher-temperature zones, for example—respond to similar exposure levels. Geographic diversity among the studies was notable, with nine based in China and one each from Thailand and the USA, collectively encompassing 3,510,530 influenza cases. Additionally, five mechanistic studies were included in a narrative overview to provide a broader understanding of underlying biological pathways. More specific details on each study and their subgroup analyses are outlined in Table 1.

**Table 1:** Studies included in the meta-analysis investigating for the relationship between PM_2.5_ and Influenza.

Reference	Time period	Study design	Study location	Population exposure	Influenza counts	Lag (days)	Ages	Confounders
(28)	2013–2014	RE	China	All	76,902	2, 3, 2–3	All	T, RH, AP, WS, HS, C, M, H, Y
(20)	2005–2016	CC	USA	All	3,246	0, 0–3, 0–6	All	G, A, RA, E, Y, S, HP, ED, P, OAP, T, RH
(30)	2011–2021	TS	Thailand	All	84,075	31	All	P,T,RH,C, H,D,R, Y, M
(48)	2013–2016	GAM	China	<15	2,569,021	2, 1	<15	HP, A, OAP, T, RH, C, H
(29)	2013–2015	R	China	All	NA	7, 14	All	OAP, T, RM, AP, ILI,
(24)	2010–2019	GAM	China	All	206	0, 1, 2, 3, 4, 5, 6, 7, 8, 9, 10, 11, 12, 13, 14	All	A, G, Y, IVA, IVB, OAP, T, AP, RH, WS, PR, AQI, IVA, IVB, ILI, AECOPD, RD
(21)	2013–2015	DL	China	All	206	4	All	IVA, IVB, OAP, T, RH, WS, HS, C, H
(25)	2015–2017	GAM	China	All	11,485	0, 1, 2, 0–1, 0–2	All	OAP, T, RH
(22)	2014–2031	DL	China	<17	16,176	0, 0–1, 0–2, 0–3, 0–4, 0–5, 0–6, 0–7	All, 0–17, 18–64, > 65	A, G, T, RH, WS, VP, OAP, S
(23)	2014–2017	DL	China	All	15,312		All, 0–6, 7–17, 18–64, > 64	A, G, T, RH, OAP, H, M, C, S
(27)	2013–2019	RF	China	<19	191,846	0, 1, 2, 3, 4, 5, 6, 7, 0–6	<19	A, G, EL, T, AP, WS, RH, HS, S

Study Design: TS, Time-Series; CC, Case Crossover; GAM, Generalized additive model; DL, Distributed Lag; RF, Random Forest; R, Regression; RE, Random Effects. Confounders: G, Gender; A, Age; RA, Race; E, Ethnicity; Y, Year; M, Month; S, Season; T, Temperature; RH, Relative Humidity; AP, Air pressure; WS, Windspeed; HS, Hours of Sunshine; PR, Precipitation; AQI, Air Quality Index; C, Cold; M, Mild; H, Hot; D, Dry; R, Rainy; OAP, Other Air Pollutants; HP, Hospitilaizations; ED, Emergency Department; P, Pneumonia; ILI, Influenza-like Illness; IVA, Influenza A; IVB, Influenza B; AECOPD, acute exacerbation of chronic obstructive pulmonary disease; RD, Respiratory Disease; EL, Education Level.

### Results of the 11 association studies

**Figure 2:**
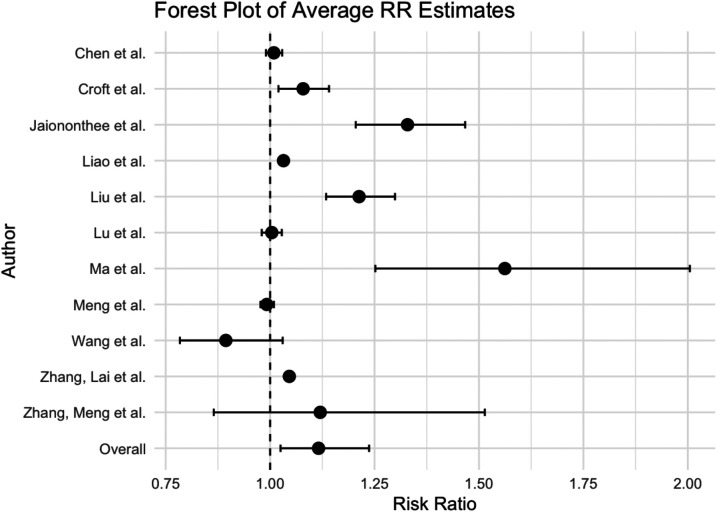
Forest plot of meta-analysis of the effect on the risk of influenza associated with PM_2.5_.

The overall weighted averaged pooled RR (Figure 2) along with the meta-analysis RR revealed that for each 10 μg/m^3^ increase in PM_2.5_, the risk of influenza increased by 11.6% and 1.5%, respectively (averaged RR = 11.6 95% CI: 2.5%, 23.7%; weighted meta-analysis RR = 1.5 95% CI: 0.8%, 2.2%). The observed difference between the overall weighted average pooled RR (11.6%) and the meta-analysis RR (1.5%) arises from differences in calculation methods. Specifically, the weighted average pooled RR was derived by directly averaging reported RRs from individual studies, weighted by study size or precision, while the meta-analysis RR was calculated using a more robust statistical approach, accounting explicitly for between-study heterogeneity and precision (e.g., random-effects meta-analysis). Consequently, the meta-analysis RR provides a more conservative and statistically rigorous estimate of the association. It is important to note that the heterogeneity of results was extremely high among the studies (Table 2). The I^2^ statistic indicated that approximately 91.90% of the variability in effect estimates can be attributed to heterogeneity rather than random chance, and the estimated amount of total heterogeneity *τ*² was calculated to be 0.0014. This high heterogeneity implies that the observed association between influenza and PM_2.5_ might vary widely across different factors not explicitly accounted for in the analysis. Variations in study design, such as the geographical location, population demographics, and seasonality, as well as differences in analytical methods—like adjustments for confounders and the specific models used to estimate risk—can all influence the observed effect sizes. For instance, studies using different definitions of influenza cases or various lag periods between PM_2.5_ exposure and influenza outcomes may yield varying risk estimates. Subgroup analyses highlighted that factors such as season, age groups, and gender had notable impacts on heterogeneity (*p* < 0.05), which underscores that these design and analytical choices affect the robustness and comparability of results.

**Table 2:** Subgroup pooled analysis for the heterogeneity factors in the included articles. Relative Risk (RR), *χ*^2^ the Q statistic-based test and *I*^2^ statistic bolded values indicate confidence intervals that do not overlap 1.

Subgroup	Included study	Sub-studies	RR (95% CI)	2-sided *p*-value	Heterogeneity measurements		
					*χ* ^2^	*I* ^2^	*P*-value
Influenza counts							
<1 k	(21, 24)	51	0.997 (0.993, 1.001)	0.134	56.167	0.27%	0.2549
1 k–10 k	(20, 22, 23)	45	1.014 (0.983, 1.045)	0.366	27.025	2.11%	0.9794
11 k–25 k	(20, 22, 23, 25, 28)	75	0.981 (0.971, 0.992)	0.0003	136.003	0.21%	<.0001
30 k–90 k	(22, 28, 30)	29	1.036 (0.971, 1.101)	0.268	87.725	94.03%	<.0001
100 k+	(27, 48)	48	**1.044** (**1.036, 1.053)**	<.0001	1,552.790	86.85%	<.0001
Age (years)							
<18	(22, 23, 48)	27	0.879 (0.780, 0.979)	0.015	123.682	98.82%	<.0001
18–64	(22, 23, 27)	65	**1.040** (**1.029, 1.051)**	<.0001	1,570.393	97.37%	<.0001
All ages	(20–24, 28, 30, 49)	136	0.998 (0.994, 1.002)	0.317	204.694	0.27%	0.0001
Season							
All temps	(20–25, 27–30, 48)	214	**1.012** (**1.005, 1.019)**	0.0001	2,225.552	92.77%	<.0001
Hot	(23, 28, 30)	11	**1.294** (**1.078, 1.509)**	0.006	25.928	58.07%	0.0038
Cold	(23, 28–30, 48)	16	**1.142** (**1.035, 1.249)**	0.008	48.926	72.55%	<.0001
Lags							
0–14	(20, 22–25, 27–29, 48)	238	**1.013** (**1.006, 1.020)**	0.0002	2,251.381	92.03%	<.0001
0–1	(20, 22, 24, 25, 27, 29, 48)	34	**1.023** (**1.009, 1.038)**	0.002	238.512	93.61%	<.0001
2–3	(23–25, 27, 28, 48)	90	1.008 (0.994, 1.020)	0.218	395.095	86.85%	<.0001
4–7	(20–22, 24, 27)	91	**1.017** (**1.003, 1.030)**	0.012	1,123.624	95.27%	<.0001
8–31	(24, 30)	33	0.998 (0.992, 1.005)	0.580	97.085	1.14%	<.0001
Overall		248	**1.015** (**1.008, 1.022)**	<.0001	2,308.679	91.90%	<.0001

Our analysis indicates that the risk of influenza due to PM_2.5_ exposure varies with seasons, showing stronger risk estimates during warmer flu seasons RR = 29.4% (95% CI: 7.8%, 50.9%) and colder seasons RR = 14.2% (95% CI: 3.5%, 24.9%). Heterogeneity among studies within a particular temperature group was moderate, 58.07% and 72.55%. Additionally, focusing on the 18–64 age group, we observed a slight increase in influenza risk due to PM_2.5_, RR = 4% (95% CI: 2.9%, 5.1%), with the heterogeneity across studies being non-significant. Of the studies that showed significant heterogeneity, the subgroup analysis by influenza counts demonstrated that larger populations (100K+) yielded higher risk estimates than smaller populations: RR = 4.4% (95% CI: 3.6%, 5.3%) per 10 μg/m^3^ increase in PM_2.5_, respectively.

### Publication bias

Publication bias results from the regression test for funnel plot asymmetry revealed a *z*-value of 1.6282 with a corresponding *p*-value of 0.1035. Although this *p*-value exceeds the conventional cutoff for statistical significance (0.05), suggesting no significant funnel plot asymmetry, the limited number of studies included reduces the statistical power of this test. Therefore, the possibility of publication bias cannot be completely excluded, and findings should be interpreted with caution (see Supplementary Figure S1). Future studies with larger sample sizes would help provide more robust evaluations of publication bias.

### Mechanistic effects

There were five studies that delved into the mechanistic underpinnings of the association between PM_2.5_ exposure and influenza (10, 31–34). Hsiao et al., identified certain chemicals within PM_2.5_ that are correlated with the presence of active influenza viruses, illuminating a potential chemical process that might facilitate the transmission of influenza. This research suggests that the chemical constituents of PM_2.5_ could play a crucial role in enabling these particles to act as carriers or vectors for the influenza virus (33). Kumar et al. found that exposure to PM_2.5_ can lead to failure to sustain optimal levels of interleukin 22 (IL-22), coupled with an inability to induce IL-22 production during flu infection. This inadequacy in maintaining IL-22 levels was proposed as a catalyst for aberrant epithelial repair and compromised immune responses, potentially escalating flu severity (31). Moreover, a different study elucidated the impairment of pulmonary immune defense mechanisms and lung tissue cell injury due to prolonged PM_2.5_ exposure, attributing increased post-infection death rates to the resultant downregulation of immune defenses (32). Additionally, findings from another investigation highlighted how PM_2.5_ compromised innate immune responses, rendering individuals susceptible to influenza by suppressing critical factors like the NLRP3 inflammasome and interferon-beta (IFN-β) expression (10). Lastly, a study exploring wood smoke particles during wildfires revealed dynamic changes in pulmonary macrophage and T-cell subsets in response to WSP and influenza challenge, providing insights into the nuanced immune response alterations triggered by these environmental factors during viral infection (34).

## Discussion

### Key findings

Our meta-analysis highlights the association between increased PM_2.5_ exposure and heightened influenza risk, with an overall 1.5% increase in relative risk (RR) per 10 µg/m^3^ increment of PM_2.5_. This relationship is significant but requires nuanced interpretation, especially given the high heterogeneity across studies. Our findings demonstrate the necessity of exploring factors beyond pooled averages, considering subgroup analyses that reveal the differential impact of PM_2.5_ across diverse populations. However, several limitations warrant further discussion, particularly concerning the subgroup analyses. While these analyses contribute valuable insight, the potential influence of confounding factors—such as socioeconomic status, baseline health, or lifestyle variations—is often under-addressed, risking overinterpretation of observed subgroup effects.

High variability exists among the included studies in terms of both study context and design. Variations in subgroup results could stem from factors other than PM_2.5_ exposure, such as underlying health status, access to healthcare, or differences in exposure measurement techniques. While the study stratifications—based on age, gender, temperature, and seasonal factors—provide compelling findings, they also introduce complexities that require careful consideration to avoid attributing all observed differences solely to PM_2.5_. Additional exploration of these confounders is essential to accurately interpret the observed associations. Potential limitations related to existing data sources were also identified, including inconsistencies in air pollution measurement techniques, variability in influenza reporting methods, and biases inherent in meta-analytic approaches. These limitations underscore the need for standardized methodologies and more consistent data collection practices in future research to ensure comparability and enhance the robustness of conclusions. Below, we discuss these findings in the context of each sub analysis grouping and their possible linkages to the impact of PM_2.5_ exposure on compromised immune response to influenza.

### Short- and long-term Pm_2.5_ exposure periods affects influenza risk

Our meta-analysis revealed that nearly all lag periods of PM_2.5_ exposure influence influenza risk. We examined various lag periods, from immediate (“0–1” day) to extended (“8–31” days), to understand the temporal relationship between PM_2.5_ exposure and influenza risk.

For the short lag periods (“0–7” days), we observed an increase in influenza risk, suggesting an almost immediate effect of PM_2.5_ on influenza susceptibility. These observations align with previous research linking PM_2.5_ exposure to increased influenza risk and respiratory issues within a week of exposure (24, 25, 27–29, 35, 36). Mechanistic studies shed light on this relationship, showing that short-term PM_2.5_ exposure can trigger interleukin-6 (IL-6) synthesis, enhancing early immune response (32). However, short-term (<7 days) exposure can lead to reduced IL-6 production, subsequently compromising the immune response, and diminishing influenza resistance in mice (32). Further research indicates that PM_2.5_ can impede the production of critical immune factors like IL-1β and interferon-beta (IFN-β), potentially aggravating flu infections and suppressing certain immune pathways (10). Additionally, Kumar et al. (31) highlighted that while initial PM exposure increased IL-22 expression in the lungs, short-term exposure or subsequent influenza infection in PM-exposed mice resulted in reduced IL-22 expression, potentially contributing to aggravated lung injury during influenza infection (31).

The highest influenza risk was noted during the longer PM_2.5_ lag periods (“4–7” days). This could suggest a more pronounced impact of prolonged PM_2.5_ exposure on the respiratory system and immune response. We did look at a longer lag effect (7–14 days) which was significant; however, due to the small number of studies looking at this period of time, we did not include it in the final analysis. Mechanistic insights into extended exposure periods indicate that PM_2.5_ might compromise the immune system, rendering individuals more susceptible to influenza later. For instance, Ma et al. indicated that prolonged PM_2.5_ exposure diminished the expression of IL-6 and IFN-β in pulmonary macrophages, reducing resistance to influenza viruses (32). Another study highlighted a decrease in the production of IL-1β and IFN-β following PM_2.5_ exposure (10). Other studies with longer correlations emerged in Montana, USA, between elevated PM_2.5_ levels during the wildfire season and increased influenza activity in the subsequent season, suggesting a potential immune suppression pathway due to prolonged exposure (9). Another study by Orr et al. found that residents in Seeley Lake, Montana, USA, had decreased lung function after prolonged exposure to PM_2.5_ from wildfires long after the initial exposure event (37). These insights demonstrate the complexity of PM_2.5_'s influence on influenza transmission, showing that it is affected by exposure duration, timing, and environmental events like wildfires, thus emphasizing the multifaceted factors influencing influenza risk in the presence of PM_2.5_.

### Impact of seasonality on flu and Pm_2.5_ exposure in temperate and tropical locations

The influence of geographic locations, specifically temperate vs. tropical regions, on influenza patterns is well-established, with differences in environmental conditions leading to distinct viral transmission dynamics that vary with climatic conditions (23, 28–30, 38). Temperate zones experience seasonal flu peaks with onsets occurring after dry and cold periods (8, 39). In some tropical regions, influenza epidemics are reported during wetter periods, while others similar regions experience year-round peaks or do not exhibit clear seasonal patterns (40, 41). However, our meta-analysis revealed a disparity in the geographic distribution of studies, with most of the studies in tropical to subtropical areas. Our findings show that seasonal flu risks are heightened during warmer seasons, a trend likely influenced by the higher temperatures and humidity in tropical regions. These findings underscore the importance of developing region-specific flu prevention strategies, considering the continuous presence of the virus in tropical regions and the seasonal peaks in temperate zones.

The relationship between temperature, air pollution, and the immune response to influenza is complex and varies across different demographic groups and environmental conditions. High temperatures, particularly when combined with air pollutants like PM_2.5_, have been shown to significantly affect susceptibility to influenza. This impact is notably pronounced in certain demographic groups, including individuals aged 18–64 years. The interaction of high temperatures with pollutants such as ozone also exacerbates respiratory issues, as indicated by multiple studies (21, 22, 42).

In colder climates, particularly during winter, the combination of lower temperatures and decreased humidity is conducive to the stability and transmission of the influenza virus (43). These environmental conditions not only facilitate the virus's survival but also may impair immune responses, increasing vulnerability to influenza (44). Additionally, during cold periods, there is an increased likelihood of people remaining indoors near one another, which further enhances the transmission of the virus. Warm temperatures, conversely, present a more complex scenario in terms of influenza impact. While warm weather might reduce the stability and transmission of the influenza virus, studies by Zhang, Meng et al. and Shin et al. indicate a complicated relationship between temperature, air pollution, and flu susceptibility in these conditions (23, 36). In warmer climates, the role of air pollution becomes more significant in influencing respiratory health, which might affect influenza transmission dynamics.

### Effect of study system and design

Our results demonstrated that factors such as population size, and age-specific analyses can influence the observed strength of association between PM_2.5_ exposure and influenza risk. Regarding influenza counts, larger populations often provide more robust and reliable statistical associations. This is a well-established principle in epidemiological studies, reflected in our findings of stronger inferences in studies with larger influenza counts aligning with existing research norms (45).

Our age-specific analyses showed a significant impact of PM_2.5_ exposure on influenza risk predominantly in the 18–64 age group, showing a reduced risk for individuals under 18. Intriguingly, no significant effect was observed in those over 64, as indicated by the relative risk value of 0.937 (95% CI: 0.887, 0.987). This absence of a noticeable effect in the elderly population, typically considered highly vulnerable to influenza, raises important questions. It could be hypothesized that this group may have reduced exposure to outdoor air pollution due to limited mobility or spending less time outdoors. Furthermore, the elderly might benefit from more robust public health interventions, like increased vaccination rates or better access to healthcare, which could mitigate the impact of PM_2.5_ on influenza risk.

For the 18–64 age group, factors unique to this demographic, such as increased periods spent outdoors, leading to higher exposure to pollution, and lifestyle characteristics that may contribute to elevated risk. Additionally, underlying health conditions that are more prevalent in this demographic might amplify the effects of PM_2.5_. Further compounding this issue is the role of immune system maturity and the presence of comorbidities, both of which are more pronounced in the adult population and may enhance vulnerability to the combined effects of influenza and air pollution. Research has shown that immune responses can vary significantly with age due to immunosenescence, which is the gradual deterioration of the immune system associated with aging. This can affect how the immune system responds to infections like influenza and to vaccinations (46). Studies have also shown that immune responses in individuals with conditions like obesity and type 2 diabetes mellitus, which are more common in adults, can significantly influence the efficacy of the influenza vaccine, highlighting the complex interaction between PM_2.5_ exposure and influenza risk in this age group (47).

### Research gaps and future perspectives

Influenza remains a formidable challenge in global public health, often heightened by the looming threat of pandemics. Our meta-analysis has delved into the intricate relationships between PM_2.5_ exposure and influenza risk, shedding light on the dynamic interplay of environmental factors and their health impacts. While PM_2.5_ levels are decreasing in some parts of the world due to stringent regulations, they are concurrently rising in others, fueled by increasing human populations and climate-induced changes such as wildfires. This emphasizes the importance of addressing air pollution within influenza public health strategies.

However, notable limitations exist. Variability in air pollution measurement methods, geographic coverage, and influenza reporting practices introduce inconsistencies, complicating meta-analysis interpretations. Standardized data collection and reporting are essential for future research. Confounders such as socioeconomic status, healthcare access, and vaccination rates, which significantly affect influenza vulnerability, were inadequately addressed. Future studies should incorporate robust methodologies to manage these confounders effectively. Additionally, interactions between PM_2.5_ and other pollutants (e.g., nitrogen oxides, ozone, sulfur dioxide) that may amplify influenza susceptibility remain under-explored. Future research should prioritize comprehensive multi-pollutant analyses to clarify these interactions. Lastly, the predominance of studies from China limits the global applicability of findings. Enhanced international collaboration and geographically diverse research are essential to improve generalizability and inform effective global public health policies.

## Conclusion

From our work, a key takeaway emerges: the impact of PM_2.5_ on influenza risk is multifaceted, influenced by exposure duration, population demographics, and environmental variables. This complexity highlights the necessity of both associative and mechanistic studies. While our analysis has provided valuable insights, it also reveals the need for more comprehensive research. To address this complexity effectively, future research must adopt a balanced approach, integrating associative studies—examining epidemiological relationships—and mechanistic studies that explore underlying biological pathways. Clarifying both how and why PM_2.5_ affects influenza susceptibility will enable public health strategies to be tailored specifically to diverse global contexts, accounting for regional variations in air pollution sources, population vulnerabilities, and environmental changes. This comprehensive, dual-focused research approach is critical for developing responsive and sustainable interventions capable of reducing influenza risks across varying environmental and demographic s

## Data Availability

The original contributions presented in the study are included in the article/Supplementary Material, further inquiries can be directed to the corresponding author.
